# Validation studies for population-based intervention coverage indicators: design, analysis, and interpretation

**DOI:** 10.7189/jogh.08.020804

**Published:** 2018-12

**Authors:** Melinda K Munos, Ann K Blanc, Emily D Carter, Thomas P Eisele, Steve Gesuale, Joanne Katz, Tanya Marchant, Cynthia K Stanton, Harry Campbell, for the Improving Coverage Measurement Group

**Affiliations:** 1 Institute for International Programs, Johns Hopkins University Bloomberg School of Public Health, Baltimore, Maryland, USA; 2 Population Council, New York, New York, USA; 3 Center for Applied Malaria Research and Evaluation, Tulane University School of Public Health and Tropical Medicine, New Orleans, Lousiana, USA; 4 Independent consultant, Bend, Oregon, USA; 5 Department of International Health, Johns Hopkins University Bloomberg School of Public Health, Baltimore, Maryland, USA; 6 Department of Disease Control, London School of Hygiene & Tropical Medicine, London, UK; 7 Stanton-Hill Research, LLC, Moultonborough, North Hampshire, USA; 8 Centre for Population Health Sciences, University of Edinburgh, Edinburgh, Scotland, UK

## Abstract

**Background:**

Population-based intervention coverage indicators are widely used to track country and program progress in improving health and to evaluate health programs. Indicator validation studies that compare survey responses to a “gold standard” measure are useful to understand whether the indicator provides accurate information. The Improving Coverage Measurement (ICM) Core Group has developed and implemented a standard approach to validating coverage indicators measured in household surveys, described in this paper.

**Methods:**

The general design of these studies includes measurement of true health status and intervention receipt (gold standard), followed by interviews with the individuals observed, and a comparison of the observations (gold standard) to the responses to survey questions. The gold standard should use a data source external to the respondent to document need for and receipt of an intervention. Most frequently, this is accomplished through direct observation of clinical care, and/or use of a study-trained clinician to obtain a gold standard diagnosis. Follow-up interviews with respondents should employ standard survey questions, where they exist, as well as alternative or additional questions that can be compared against the standard household survey questions.

**Results:**

Indicator validation studies should report on participation at every stage, and provide data on reasons for non-participation. Metrics of individual validity (sensitivity, specificity, area under the receiver operating characteristic curve) and population-level validity (inflation factor) should be reported, as well as the percent of survey responses that are “don’t know” or missing. Associations between interviewer and participant characteristics and measures of validity should be assessed and reported.

**Conclusions:**

These methods allow respondent-reported coverage measures to be validated against more objective measures of need for and receipt of an intervention, and should be considered together with cognitive interviewing, discriminative validity, or reliability testing to inform decisions about which indicators to include in household surveys. Public health researchers should assess the evidence for validity of existing and proposed household survey coverage indicators and consider validation studies to fill evidence gaps.

Population-based measures of intervention coverage, defined as the proportion of individuals in need of a service or intervention who actually receive the service or intervention, are used at the country and global level to track progress in delivering high impact interventions to populations in need [1] and to evaluate the impact of large-scale health programs. Nationally representative household surveys implemented by The Demographic and Health Surveys (DHS) Program [2] and the Multiple Indicator Cluster Survey Program (MICS) supported by UNICEF [3] have been providing population-based estimates of intervention coverage in low and middle income country (LMIC) settings since the 1980s and 1990s, respectively.

The validity of survey questions is often investigated by assessing whether questions represent the items of interest (content validity), examining associations between survey items that are expected to be correlated or that are expected not to be correlated (construct validity); assessing whether survey data agree with an objective gold standard (criterion validity), or examining cognitive processes that respondents use when answering survey questions (cognitive interviewing) [4,5]. In addition, assessments of missing information and patterns of missingness and of reliability can provide information on the quality of data elicited by survey questions.

Historically, validation research on DHS and MICS MNCH intervention coverage indicators has focused primarily on content validity, construct validity, and cognitive interviewing, as well as assessments of data quality [6-10]. All of these methods provide valuable information about survey questions and data; however, they cannot quantify the extent to which survey-based measures of intervention coverage differ from the objective “truth” that they seek to measure (criterion validity). In addition, assessing construct validity of intervention coverage indicators can be challenging, as the correlation between these questions and other survey items may be unknown or may vary between settings. For these reasons, studies comparing survey responses to a gold standard measure of what actually happened are essential to understanding whether the indicator provides accurate information about country or program performance, or whether the information provided is not useful or even misleading. Until recently there have been few such validation studies for coverage indicators measured in household surveys.

The Improving Coverage Measurement (ICM) Core Group, first under the Child Health Epidemiology Reference Group (CHERG) and then under a separate grant from the Bill & Melinda Gates Foundation, has developed and implemented a standard approach to validating coverage indicators measured in household surveys by comparing them against a gold standard. The results of these studies are published in this Research Theme on improving coverage measures for Maternal, Neonatal and Child Health (MNCH) and in a previous Collection published in 2013 [11].

This paper aims to describe the approach used by ICM to validate coverage indicators in household surveys, including considerations around the study design and methods, analysis, presentation and interpretation of the results, and strengths and limitations of these studies. This approach has been developed and implemented for maternal, newborn, and child health coverage indicators, but these methods can be extended to other areas, such as nutrition and reproductive health coverage indicators.

## METHODS

Following reporting guidelines promotes transparency, quality, and completeness of reporting and facilitates information/data synthesis for the purpose of systematic reviews and meta-analyses. Validation study methods and results should present data described in the Standards for Reporting Diagnostic Accuracy (STARD) guidelines (see http://www.equator-network.org/reporting-guidelines/stard) [12]. Further guidance on reporting of studies on validity of measures for prediction of a reference value can be found in the Cochrane Collaboration Diagnostic Test Accuracy Working Group guidelines (see http://srdta.cochrane.org and Cochrane Handbook at http://srdta.cochrane.org/handbook-dta-reviews).

### General study design

**Figure 1:**
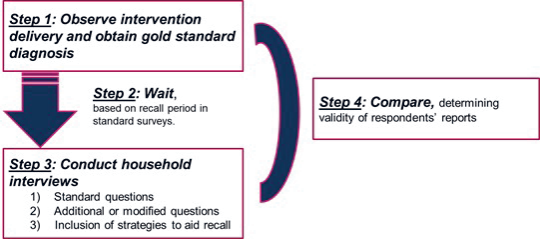
Overall design of Improving Coverage Measurement (ICM) coverage validation studies.

Studies validating coverage measures seek to compare coverage estimates obtained from representative household surveys with “true” coverage. Methods for coverage indicator validation studies are based on methods used to evaluate the validity of diagnostic tests. These approaches assess the ability of coverage measurement tools (typically, household survey questionnaires) to correctly classify an individual’s need for and receipt of an intervention by comparing the data collected using the tool against a gold standard measure. The general study design for validation of coverage indicators collected through household surveys includes 1) direct observation or measurement of true health status and intervention delivery among a sample of individuals (gold standard), 2) a recall period, preferably similar to that allowed for recall of the intervention in household surveys, 3) survey interviews with the individuals observed in Step 1, using questions worded as in the household survey, and 4) a comparison of the observations (gold standard) to the responses to the survey questions (**Figure 1**).

### Gold standard: direct observation or measurement of true health status **and** intervention delivery

The ideal gold standard would be an objective measurement of the the “truth” – ie, the true health status of an individual and whether they received the intervention in question. However, any gold standard will be subject to some degree of measurement error, depending on the intervention in question, the context, and the method used to obtain the gold standard. As a result, we cannot recommend a single gold standard, but rather recommend that investigators take the following considerations into account when selecting a gold standard:

1. Measurement error: What are the potential sources and degree of measurement error in the gold standard measure? Can they be mitigated or quantified, eg, through improved training or standardization of data collection practices? If a biomarker is used, has it been validated for this purpose?
2. Bias: Is the measurement error likely to be differential according to relevant variables, such as whether the intervention was received, participant health status/diagnosis, or education or other socio-demographic characteristrics?
3. Effect on reporting: How likely is the gold standard (including the participant enrollment process, if any) to have an effect on participant reporting or recall of health status or intervention receipt?
4. Feasibility: How feasible is the gold standard to implement across the required sample size, within a reasonable amount of time and within the available budget?

The primary approach for obtaining gold standard measures for receipt of an intervention is direct observation of clinical care. This observation is often conducted by a trained clinician, such as a nurse or nurse/midwife [13,14]. Provider medical records may also be considered the gold standard for some validation studies, if of sufficiently high quality [15], or may be used to complement other gold standard methods [16]. Validation studies for careseeking indicators may employ GPS tracking, barcoded ID cards, physical tokens, or other methods for documenting patient visits to health providers in the study area [16]. Examples of gold standards in this Collection include direct observation of clinical care, examination of health facility records, use of GPS, and bar-coded identification cards to track care-seeking, and some triangulation of several different measures.

For some coverage indicators, the determination of need (ie, the denominator) is relatively straightforward and is based on factors such as age, sex, or pregnancy status. These characteristics have not typically been validated, although reporting of age and pregnancy status are both prone to some degree of reporting error. For coverage indicators for curative interventions, however, the denominator is based on an individual’s diagnosis or reported signs and symptoms, and should be validated against a gold standard. Studies that seek to validate measures of need should aim to enroll individuals both with and without the condition in order to obtain precise measures of specificity. Where the assessment of need is complex (eg, diagnosis of pneumonia), it is desirable to employ study-trained clinicians to diagnose and manage enrolled individuals in order to ensure an accurate gold standard [17], but trained lay workers may also be used for less complex assessments, such as measuring birthweight [18].

Validation studies may also use survey interviews conducted as soon as possible after the provision of the intervention as the gold standard, under the assumption that limiting the recall period will reduce error. This approach differs from other gold standards in that it does not make use of a data source external to the survey respondent. These gold standard interviews, coupled with later follow-up interviews, are useful for assessing indicator reliability and the erosion of recall over time. However, this approach is not well-suited to evaluate the overall accuracy of an indicator, as the respondent may provide inaccurate responses in the gold standard interview. For example, the respondent may not understand, or may mis-understand, the survey question(s), leading to inaccurate responses. For this reason it is particularly important that the interviewer be well-trained, but even so, responses may be inaccurate. Respondents also may not know which intervention they received. For example, a woman may receive a uterotonic injection after childbirth but may not be told the nature or purpose of the injection. Finally, respondents may not be able to report accurately on their need for an intervention, because they were not given a diagnosis, they misunderstood their diagnosis, or they were mis-diagnosed.

If there are concerns that the gold standard for a particular study could change participant recall or behavior, the study may incorporate control groups to assess this effect and adjust for it. For example, a careseeking validation study that gave women phones with a GPS-tracking app to detect careseeking visits, with frequent follow-up visits to enrolled households, used two control groups to assess the effect of the phones and the follow-up visits. The first control group did not receive phones but still had monthly follow-up visits, while the second group did not receive phones and had only one follow-up visit.

### Recall period

Following the direct observation or measurement of health status and/or intervention delivery, time is allowed to elapse before the follow-up visit to administer the survey questionnaire. In general, individuals should be interviewed within the range of the relevant recall period employed by the household survey. Where possible, it may be beneficial to build a greater range of recall periods into the study design to assess recall decay and to determine the optimal recall period for a specific intervention. Where long recall periods (eg, 1 year or more) are used, it may also be useful to begin by evaluating a shorter recall period, and use these interim results to decide whether evaluation of a longer recall period is warranted. This was the approach used by a maternal indicator validation study in Kenya [14,19].

### Survey interviews

When designing the survey tools for coverage validation studies, attention should be paid to the wording of survey questions and to the accuracy of translation from one language to another. Survey questions (and their translations, to the extent possible) should match other existing standardized surveys. Where this is not possible – for example, where questions differ between household surveys – the intent and structure of the questions should be preserved. At the time the survey interviews are conducted, other questions may be added with the aim of improving the validity of the indicator. These may include testing new questions, prompts, or use of recall aids such as videos, photographs, or drawings of activities or treatments related to the intervention. However, it is important that these additional questions or tools only be administered after the original survey questions to avoid biasing responses. Alternately, one could randomize the order of the prompts/new questions to be able to examine whether a modification improves validity relative to the original survey question. However, this would require a larger sample size. Assessment of wording and cognition around survey questions may also be conducted [5,20] or additional probing may be used to identify reasons for incorrect responses. Examples of such studies are the testing of “pill board” photographs to improve caregiver recall of treatment for symptoms of acute respiratory infection, testing of videos of severe pneumonia to improve the accuracy of reporting of acute lower respiratory infection [17], and qualitative in-depth interviews and focus group discussions with respondents and data collectors on recall of birth outcomes in Nepal [18] and labor and delivery experiences in Kenya [19]. The survey tool should also collect information on demographic or other covariates to assess whether need for or receipt of interventions are more accurately reported among some subgroups than others, or whether interviewer characteristics influence reporting.

### Sample size

The sample size for indicator validation studies is generally determined based on the desired level of precision for sensitivity and specificity estimates. A level of precision of approximately 5-6 percentage points is usually sufficient for drawing inferences regarding the indicator while maintaining a reasonable sample size (approximately 200-400 individuals with and without the intervention). If multiple arms are included in the study – for example if different recall periods or question formulations are being tested – the sample size should ensure an adequate level of precision in each arm.

The sample size and/or sampling design should also take into account the prevalence of the intervention or behavior assessed. For example, if validating maternal recall of delivery by cesarean section, the study must ensure that sufficient deliveries by cesarean section (rare in many low income settings) are observed to obtain the desired level of precision. This can be accomplished either by increasing the total sample size for the gold standard to ensure that enough cases with and without the intervention are observed (eg, [21]), or by purposively enrolling individuals who have and have not received the intervention (eg, [17]). The former approach may be more appropriate when a study is validating multiple indicators, only some of which are rare.

In addition to precision and intervention prevalence, the sample size should account for non-response, loss to follow up, and any design effect if cluster sampling is used; the magnitude of these adjustments will vary according to the setting, indicator, and time interval between enrollment and follow up interview.

### Ethical considerations

The gold standard approaches described here, as well as the survey interviews, are considered human subjects research and require informed consent from clients and providers and ethical approval from Institutional Review Boards. Ethical issues to consider in the design of coverage validation studies are whether to intervene and how, if an observer witnesses poor quality or harmful clinical care; the education or skill level of the observer; how to develop a consent form that does not bias participant responses; and how to obtain informed consent from women who are in labor or severely ill individuals (or their caregivers).

## RESULTS

### Study participants

**Figure 2:**
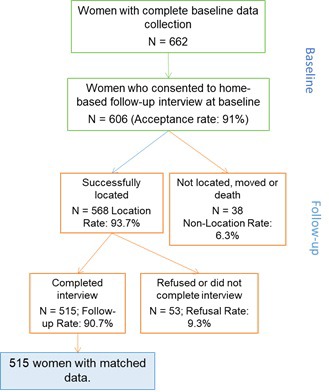
Example of flowchart of participant enrollment and follow-up (A. Blanc, personal communication, 28 December 2017).

It is important to present data on the sampling procedures, including the level of participation in any comparison groups. The results should state the number of participants eligible to be included in the study and the number included at each stage, including the gold standard measure, survey interview, and in the final analyses. For follow-up studies like these, this information is often best presented in a flowchart (**Figure 2**). The main reasons for non-participation should be given in order to assess representativeness to the underlying population. An assessment of whether missing data occur at random should be made, where possible. Without going back and surveying some of the participants with missing data, one cannot definitively identify whether data are missing at random. Scientific knowledge about the data or examining patterns in the data can help identify whether data are missing at random but this requires judgement [22].

A table should present the characteristics of the population studied so that representativeness of the study population to the underlying population can be assessed. This should generally include demographic data (eg, age, sex, ethnicity), risk factors, disease characteristics and details of any comorbidity, where appropriate. If the study includes a comparison group, the table should present the characteristics of each group and should include potential confounding factors.

### Individual-level validity

**Figure 3:**
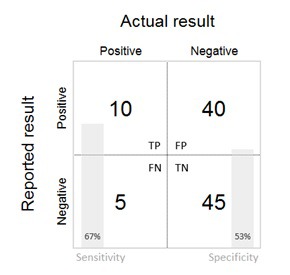
Simplified 2 × 2 table.

The validity of the survey data in measuring the parameter under study should then be presented. The results section provides data on the comparison of survey data to a gold standard. This is generally presented in terms of sensitivity, specificity and positive and negative predictive values in a summary 2 × 2 table of test (survey) positive / negative by true (gold standard) positive / negative status (**Table 1** and **Table 2**). Where appropriate and where sufficient sample size permits, stratified 2 × 2 tables can be presented by the covariates of interest. A simplified 2 × 2 table for less technical audiences may also be used (**Figure 3**). If the study is validating both the numerator and denominator of an indicator, 2 × 2 tables, sensitivity (SN), specificity (SP), and other validation metrics should be presented separately for each.

**Table 1:** 2 × 2 validation table

	Gold standard			
**Self-report (survey measurement)**		**Positive**	**Negative**	**Total**
	**Positive**	a	b	a + b
	**Negative**	c	d	c + d
	**Total**	a + c	b + d	n

**Table 2:** Definitions of common validation metrics*

Term	Definition	Formula†
Sensitivity	The proportion of individuals who truly received an intervention who were classified as having received the intervention by survey questions.	a/(a + c)
Specificity	The proportion of individuals who truly did not receive an intervention who were classified as not having received the intervention by survey questions.	d/(b + d)
Area Under the Receiver Operating Characteristic Curve (AUC)	The probability that a test will correctly classify a randomly selected set of one positive observation and one negative observation	Calculated as the area under the curve of sensitivity plotted against (1 − specificity)
Accuracy	The proportion of individuals surveyed who were correctly classified as having received or not having received the intervention.	(a + d)/n
Positive Predictive Value	The probability that an individual received an intervention, given that they reported receiving the intervention	a/(a + b)
Negative Predictive Value	The probability that an individual did not receive an intervention, given that they reported not receiving the intervention	d/(c + d)

*Adapted from [23,24].

†Variables are defined in **Table 1****.**

For interventions with very low or very high prevalence/incidence, validation may not be possible due to sample size. For example, in the studies in this Collection, validation results were not presented if there were fewer than 5 observations in any cell of the 2 × 2 tables for sensitivity and specificity of a coverage indicator. In such cases, it is important that the percent agreement between survey data and the gold standard be presented.

**Figure 4:**
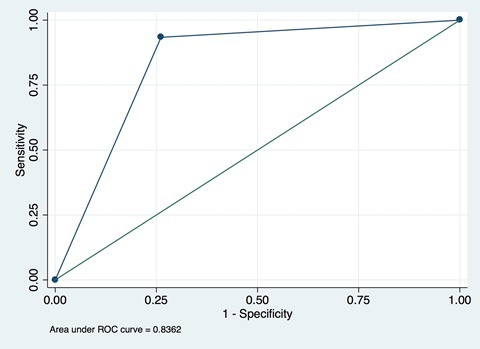
Example of area under the curve plot for sensitivity-specificity pairs.

In addition, area under the curve (AUC) can be presented. AUC is calculated as the area under the curve for a plot of SN against 1 − SP (**Figure 4**) and is useful as a summary measure of the individual validity of an indicator. AUC can take on values between 0 and 1. An AUC of 1 indicates that survey questions provide a perfect measure of whether an individual received an intervention; an AUC of 0.5 indicates that the survey questions are no better than a random guess to determine whether an individual received an intervention or did not; and an AUC of less than 0.5 indicates that the questions produce misleading information about whether an individual received an intervention – worse than a random guess. Most AUCs for coverage validation studies will fall in the range of (0.5, 1). The AUC for these types of validation studies represents the average of the sensitivity and specificity and is useful as a summary metric. If used, AUC should be presented alongside SN and SP in order to better understand the kinds of reporting bias affecting the indicator.

As noted above, percent agreement, often referred to as “Accuracy”, may also be reported. Although percent agreement is more intuitive than AUC and can be computed with small cell sizes, it varies based on the underlying prevalence of the indicator being validated, and at very high and very low prevalence levels may distort the true individual-level validity of a measure [25]. Where 2 × 2 cell sizes permit, we recommend always reporting sensitivity, specificity, and AUC as the primary metrics of individual-level validity in order to accurately reflect overall validity and understand whether an intervention is being under- or over-reported.

Comparisons of the validity of different survey questions can be made by comparing SN, SP, and AUC values. These measures of individual validity are fixed characteristics and should be invariant to the prevalence of intervention coverage in a particular setting.

These validation measures should be expressed with appropriate 95% confidence intervals, and should be presented separately for each study site in a multi-centre study.

Variation in indicator validity should be reported by participant and interviewer characteristics – including age, sex, parity, and ethnicity where relevant and available. Differences by characteristics should be tested using chi- squared tests (or Fisher exact test with cell counts <10). Associations between interviewer and participant characteristics and measures of validity can be assessed by logistic regression. In circumstances where the validity of the measure has been evaluated in several settings, these analyses may help in understanding the reasons for variation across populations. In some studies, the findings of parallel qualitative data studies on question wording may be presented in order to help interpret quantitative findings [19].

### Population-level validity

The inflation factor (IF) is the measured coverage value divided by the true coverage value in the population, and provides an estimate of the extent to which the survey-based estimate accurately reflects the true population coverage. If validation study participants were selected from the population using probability sampling, the IF can be calculated directly as the ratio of the measured coverage value in the validation study follow up interviews divided by the true population coverage value estimated from the measured sensitivity and specificity. The formula for estimating true population coverage is derived from Vecchio [26]:

*P* = Pr − (1 − *SP*) / *SN* + *SP* − 1

Where P is the true population coverage (prevalence), Pr is the measured population coverage, SN is the sensitivity, and SP the specificity. In the case where validation study participants are selected on disease status or intervention receipt, the measured coverage value must be estimated from the sensitivity and specificity estimates across a range of true coverage values, using the following equation from Vecchio [26]:

Pr = P (*SN* + *SP* − 1) + (1 − *SP*)

**Figure 5:**
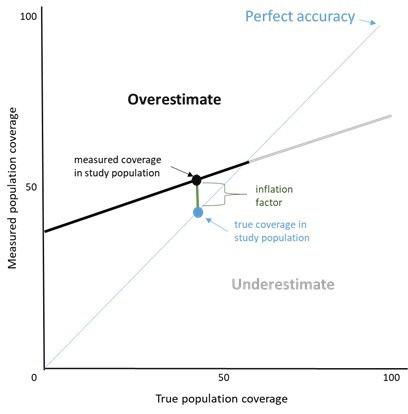
Inflation factor scatterplot.

The IF represents the population-level validity of the measure and varies by the coverage prevalence in the population. The IF should be presented along with the true coverage and survey-based estimate of coverage in the study population. In addition, estimates of measured coverage should be plotted over a range of true underlying population coverage levels. An example of this is given in **Figure 5** and shows that, at differing true coverage levels, respondent report could either over-estimate or substantially under-estimate the true coverage of the indicator (the Stata code for generating this graphic is given in Appendix S1 of **Online Supplementary Document(Online Supplementary Document)**) (Stata Corp, College Station, TX, USA). A graphic depicting the impact of prevalence on IF is used in multiple studies in this Collection.

### “Don’t know” responses and missing data

The percent of survey responses that are “don’t know” or missing should always be reported. Variables with a high proportion of don’t know or missing responses should be flagged as being of concern for use in surveys, unless well documented statistical approaches to imputing missing data can be demonstrated.

## DISCUSSION

It is vital that implementers, governments, and donors are able to trust the coverage measurement estimates that inform investment and programming decisions for priority interventions in reproductive, maternal, newborn, child and adolescent health [27]. These estimates are useful only to the extent that they accurately reflect true population coverage across the settings in which they are used. Thus, evidence about the validity of coverage data collected through household surveys across different settings, generated through rigorous and transparent validation studies, is essential. To enhance the knowledge base on methods for validating survey-based coverage indicators, this manuscript has described a general approach to designing validation studies that use a gold-standard measure. Here we discuss the strengths, limitations, and implications of these methods.

When reporting validation study findings, the study characteristics and their implications for interpretation of results need to be carefully documented, and potential limitations identified and discussed. A major strength of these methods is that they allow survey-based, respondent-reported coverage measures to be validated against more objective measures of need for and receipt of an intervention (the gold standard). However, all gold standard measures are subject to some degree of error. Publications and reports presenting validation results should include both a detailed description of the procedures for obtaining the gold-standard measure, and a reflection on the limitations of these procedures and their possible influence on results. The act of enrolling participants into the study and observing clinical care may influence individuals’ recollection of the care received. Medical records are not always complete or accurate, and information solicited from providers on content of a health service encounter may be subject to recall and social desirability bias. Nevertheless, events observed or reported by a health provider are often the best available source to create the gold standard against which to validate survey measures of coverage.

The design phase of validation studies should consider the likely limitations in current measurement practice and aim to produce results that can provide a way forward: not only to identify measures with high or low validity but also where possible to propose improved measurement methods. For example, by using well-established survey questions alongside new adaptations (eg, probes or memory prompts) the potential added value of a change to current wording may be understood. If the measurement method is constrained by a period of recall (for example because of sample size considerations) then testing a range of different recall periods, including the standard practice, can assist decision-making about future measurement approaches. As part of this plan the analytical protocol, including definitions and cut-offs, should be defined and align where possible with existing evidence or clearly state where and how definitions differ.

Most validation studies seek to inform recommendations about whether an indicator should be measured in household surveys. The AUC and IF are generally used for this purpose; however, there are no standards for what constitutes an acceptable level of individual or population-level bias. Earlier coverage indicator validation studies funded by ICM and the CHERG defined cutoffs for individual-level accuracy as high (AUC>0.70), moderate (0.60<AUC<0.70), and low (AUC<0.60), and population-level bias as low (0.75<IF<1.25), moderate (0.50<IF<1.5) and large (IF<0.50 or IF>1.5) [3,5,6]. Based on a further review of validation study results, ICM currently uses cut-offs of AUC≥0.70 AND 0.75<IF<1.25 for inclusion of an indicator in a large survey programme, with lower cut-offs (AUC≥0.60 OR 0.75<IF<1.25) for specialized, in-depth surveys. However, these criteria are by nature arbitrary, and higher or lower thresholds could be justified. Despite acknowledged limitations to the use of AUC and IF for indicator validation, the ICM Core Group strongly recommends the use of both because of the individual and population-level perspectives they provide. Individual-level and population-level validity should be considered together when making decisions about which indicators to measure; both should be at acceptable levels for an indicator to be added to a survey programme.

For coverage indicators in which the denominator requires validation (eg, curative interventions), both the numerator and denominators must meet the study’s pre-established thresholds. For rare conditions, such as pneumonia, it is important to consider the potential number of false positives given the specificity and true prevalence of the condition. If the survey questions result in many false positives in the denominator, the indicator will be misleading, even if the numerator can be accurately measured.

The context in which the validation study is conducted should also be taken into account in the interpretation of the results. As noted above, the IF will depend on the prevalence of the coverage indicator in the study setting, and it is likely that individual-level validity also varies somewhat between settings. For example, a validation study conducted in a relatively homogenous rural population may not produce the same results when applied in a more heterogeneous, urban setting, and the interpretation should reflect this. Similarly, in settings where careseeking is extremely low, validation studies using provider-client interactions or medical records as the gold standard may produce biased validity measures compared to the larger population in need of the intervention. For this reason, it is generally desirable to conduct validation studies in a range of settings before making a firm recommendation about the inclusion of an indicator in household surveys.

## CONCLUSION

The careful tracking of the coverage of high impact health interventions is central to decision making around the investment of resources and around evaluating the impact of these investments on populations in need. There is increased attention to accountability regarding health care resources; however the current state of routine health information system data remains variable [27-30] and a substantial percent of the population does not access care when needed from a public health facility. Household surveys, in which adult women are asked to report on their own experience and that of their children, remain the most obvious option for the collection, analysis, and dissemination of intervention coverage data. The mandate for the ICM project has been to increase the amount and quality of the evidence around the validity of coverage indicators for high impact MNCH interventions.

The intention of this paper is to describe a methodology that we hope will contribute to higher quality and greater standardization of validation studies of intervention coverage measures – their design, analysis, and interpretation. The recommendations result from our experience over the last 7 years conducting 10 validation studies in diverse settings [13,16-19,21,28,29] thus not every study follows all recommendations presented here. For example, earlier studies 1) used the term “reference standard” vs our now recommended “gold standard”, accepting that any gold standard will have limitations; 2) used a cut-off of AUC≥0.6, which we decided was too low and increased to AUC≥0.7; 3) did not always publish the percent of don’t know responses; 4) did not present the results for the percent agreement between respondent reports and the gold standard for indicators for which validation results could not be presented due to sample size; and 5) used a variety of different graphics to illustrate results vs the graphs provided in this paper. Although there is no consensus on an acceptable level of accuracy for coverage indicators, we hope that using a more standardized methodology for indicator validation will improve the generalizability of results and lead to the development of alternative indicators, improved question wording, or innovations in data collection (eg, biomarkers). We note that most of the studies cited here focused on interventions delivered primarily through health facility settings; validation of home-based interventions presents additional design challenges, particularly around obtaining an appropriate gold standard. Evidence from indicator validation studies should be taken in conjunction with the results of other assessments such as cognitive interviewing, discriminative validity, or reliability testing to inform the interpretation of indicators and decisions about whether indicators/questions should be included in a particular survey or the appropriate length of recall.

Studies conducted using this methodology have led to a number of insights. For example, the proportion of children with symptoms of acute respiratory infection whose caretaker reports receiving antibiotic treatment has been found to be a poor proxy for the proportion of children with pneumonia who received antibiotic treatment in studies in Pakistan and Bangladesh [17,29]. Other studies that examined intrapartum and immediate postnatal interventions had mixed results, but generally found that women’s recall of many interventions that take place during and immediately following a delivery, especially those that require reporting on timing (eg, breastfeeding within the first hour) or sequence (eg, uterotonic given before or after delivery of the placenta) have low levels of validity, although standard indicators like cesarean section tended to be better reported [13,19,21]. Interventions received during postnatal care visits seem to be more accurately reported [30]. Indicators of preterm birth and low birthweight based on mothers’ recall underestimated these indicators in Nepal [18]. On the other hand, a study of maternal recall of care-seeking for childhood illness in rural Zambia found that mothers’ reports were valid [16].

The studies in this and the previous Collection assess the validity for a range of coverage indicators for maternal, newborn, and child health. However, relatively few other health interventions reported on in household surveys have been the subject of validation studies. For example, to our knowledge, women’s reporting of the content of family planning counseling has not been validated. Few (if any) nutrition coverage indicators, such as vitamin A or iron-folic acid supplementation, or newborn health interventions, have been assessed for validity. As maternal, newborn, and child mortality rates fall and more attention is devoted to interventions for adolescent health and non-communicable diseases, it is likely that additional indicators and questions will be added to DHS and MICS. Public health researchers in these and other global health domains should assess the evidence for validity of existing and proposed survey-based coverage indicators and consider conducting validation studies to fill the evidence gaps for indicators that have a viable gold standard for comparison purposes. Assessing the validity of respondent reports in household surveys is a key component of improving the quality of the evidence used in planning, policy-making, and evaluation.
