# Supplementary material for: Validation studies for population-based intervention coverage indicators: design, analysis, and interpretation
Source: J Glob Health. 2018 Sep 6;8(2):020804. doi: 10.7189/jogh.08.020804 (PMC6126515; doi:10.7189/jogh.08.020804)
Supplement: Online Supplementary Document [file jogh-08-020804-s001.pdf]

Online Supplementary Document

Munos et al. Validation studies for population-based intervention coverage indicators: design, analysis, and interpretation

J Glob Health 2018;8:020804

How to use IF_graph.do

After running the "IF_graph.do" you can call any of the three programs.

IF_graph_i
IF_graph_single
IF_graph_multi

Ways to use:

1. Plug in values directly:

   If you want to create a graph for a single indicator you can reference the values directly in the program call-line. You can run this command without loading a dataset in Stata.

   Values must be specified in decimal format and in the order 1) sensitivity, 2) specificity, 3) true coverage in population

   Example:

   IF_graph_i 0.8 0.75 0.52

   This code would correspond to an indicator with:
   Sensitivity = 0.8
   Specificity = 0.75
   True coverage = 0.52

2. Single indicator in a dataset:

   To create a graph for a single indicator, you can reference the values in a loaded dataset in Stata. The dataset must include variables with the sensitivity, specificity, and true coverage value already calculated. The command cannot calculate the sensitivity etc from multiple dichotomous observations.

Variables must be specified in the order 1) sensitivity, 2) specificity, 3) true coverage in population. Value stored in variable must be in decimal format.

IF_graph_single sens spec true_cover

This code would correspond to an indicator with:
Sensitivity = sens
Specificity = spec
True coverage = true_cover

3. Multiple indicators or stratified indicator in a dataset:

   To create a graph for multiple indicators or the same indicator stratified by another variable (country, etc) you can reference the values in a loaded dataset in Stata. The command is similar to "IF_graph_single" but with an additional "by" variable that can be used to specify the variable to use in the stratification. Only one sensitivity, specificity, and true coverage variable can be specified. The dataset must include variables with the sensitivity, specificity, and true coverage value already calculated.

   Variables must be specified in the order 1) sensitivity, 2) specificity, 3) true coverage in population, 4) "by" variable. Values stored in variable must be in decimal format. <u>The "by" variable must be in integer form and include value labels.</u>

   IF_graph_multi sens spec true_cover country

   This code would correspond to:
   Sensitivity = sens
   Specificity = spec
   True coverage = true_cover
   "By" = country

sens	spec	true_cover	country
0.8	0.72	0.6	Kenya
0.82	0.88	0.67	Malawi
0.65	0.9	0.58	Tanzania
0.9	0.2	0.67	Zambia
0.7	0.54	0.45	DRC

```
***************************************************************************************
*GRAPH OF PREDICTED VERSUS TRUE COVERAGE*
***************************************************************************************

*PROGRAM: IF_graph_multi
*Generates graph of predicted versus true coverage using existing dataset for multiple indicators or countries
*Requires 4 variables to be specified: 1 sensitivity, 2 specificity, 3 true coverage, 4 "by" variable
*By variable must consist of integers, cannot exceed 10 values, must have value labels
*Code example: IF_graph_multi sens spec prev country


capture program drop IF_graph_multi
program define IF_graph_multi

quietly {
    preserve

    *create by variable in correct format (0-9)
    bysort `4': gen id2=_n // sort on by variable
    egen id3 = rank(`4') if id2==1 // order by variable through rank
    gen id = id3-1 // create by id starting at 0

    sum id
    local maxx= r(max) // extract maximum by val for range

    *calculate predicted coverage over range of true coverage (0-1) in increments 0.1
    foreach k of num 0/`maxx' {

    foreach i of num 0/10 {
    gen prev`k'_`i' = (`i'/10) // true coverage - x var for predicated coverage line
    gen ifif`k'_`i' = ((`i'/10) * `1') + (( 1 - (`i'/10)) * (1 - `2')) if id==`k' // predicted coverage based on sens and spec - y var for predicated coverage line

    gen xxxx`k'_`i' = (`i'/10) // x var for 45 degree line
    gen yyyy`k'_`i' = (`i'/10) // y var for 45 degree line

    }
    }

    keep if id2==1 // keep only one observation per by variable

    *generate list of new variables for reshape
    unab mylist: prev*_1 ifif*_1 xxxx*_1 yyyy*_1

    foreach v of local mylist {

    local stubs `"`stubs' `=substr("`v'",1,6)'"'

    }

    *reshape new variables to long by id
    reshape long `stubs', i(id) j(index)

    *calculate difference between estimated coverage and 45 degree line at true coverage level in population
    foreach k of num 0/`maxx' {

    gen obsx`k' =  `3' if id==`k' // true coverage in pop
    gen obsy`k' = (`1' * `3') + ((1 - `3') * (1 - `2'))  if id==`k' // predicted coverage at true coverage in pop
    replace obsy`k' = `3' if index<5 & id==`k' // additional y point needed for scatter plot

    *label graph variables using "by" variable labels
    local lbe : value label `4'

    gen num`k' = `4' if id==`k' // create variable to link id var and "by" var
    sum num`k'
    local numm`k' = r(max) // create local with value on new var
```

```
    local f`k' : label `lbe' `numm`k''
    label var ifif`k'_ "Measured Coverage: `f`k'' "
    label var obsy`k' "Difference: `f`k'' "

    }

    label var xxxx0_ "Perfect Measure"

    *create empty variables for unused "by" levels up to 9
    local ct= `maxx'+1

    foreach k of num `ct'/9 {

    gen ifif`k'_ = .
    gen prev`k'_ = .
    gen obsx`k' = .
    gen obsy`k' = .

    }

    *create local that will be used to display only used"by" levels in legend
    local ct2= ((`maxx'+1)*2)+1

    foreach k of num 1/`ct2' {

    local O`k' = `k'

    }

    *generate graph
    twoway line xxxx0_ yyyy0_ , lcolor(gray) lp(dot) || ///
        line ifif0_ prev0_ , lcolor(red)|| scatter  obsy0 obsx0, connect(stairstep) mcolor(red) lc(red) || ///
        line ifif1_ prev1_ , lcolor(blue)|| scatter  obsy1 obsx1, connect(stairstep) mcolor(blue) lc(blue) || ///
        line ifif2_ prev2_ , lcolor(green)|| scatter  obsy2 obsx2, connect(stairstep) mcolor(green) lc(green) || ///
        line ifif3_ prev3_ , lcolor(purple)|| scatter  obsy3 obsx3, connect(stairstep) mcolor(purple) lc(purple) || ///
        line ifif4_ prev4_ , lcolor(orange)|| scatter  obsy4 obsx4, connect(stairstep) mcolor(orange) lc(orange) || ///
        line ifif5_ prev5_ , lcolor(teal)|| scatter  obsy5 obsx5, connect(stairstep) mcolor(teal) lc(teal) || ///
        line ifif6_ prev6_ , lcolor(navy)|| scatter  obsy6 obsx6, connect(stairstep) mcolor(navy) lc(navy) || ///
        line ifif7_ prev7_ , lcolor(pink)|| scatter  obsy7 obsx7, connect(stairstep) mcolor(pink) lc(pink) || ///
        line ifif8_ prev8_ , lcolor(red)|| scatter  obsy8 obsx8, connect(stairstep) mcolor(red) lc(red) || ///
        line ifif9_ prev9_ , lcolor(yellow)|| scatter  obsy9 obsx9, connect(stairstep) mcolor(yellow) lc(yellow) ///
        ylabel(0(0.2)1, gmin angle(horizontal)) ytitle("Measured Coverage") ///
        xlabel(0(0.2)1) xtitle("True Coverage")  aspectratio(1) graphregion(color(white)) legend(position(3) cols(1) ///
        size(small) order(`O1' `O2' `O3' `O4' `O5' `O6' `O7' `O8' `O9' `O10' `O11' `O12' `O13' `O14' `O15' `O16' `O17' `O18' `O19' `O20' `O21' ))

    restore

}
end


**********************************************************************************

*PROGRAM: IF_graph_single
*Generates graph of predicted versus true coverage using existing dataset for one indicator or country
*Requires 3 variables to be specified: 1 sensitivity, 2 specificity, 3 true coverage
```

```
*Code example: IF_graph_single sens spec prev


capture program drop IF_graph_single
program define IF_graph_single

quietly {
	preserve

	*calculate predicted coverage over range of true coverage (0-1) in increments 0.1
	foreach i of num 0/10 {

	gen prev_`i' = (`i'/10) // true coverage - x var for predicated coverage line
	gen if_`i' = ((`i'/10) * `1') + (( 1 - (`i'/10)) * (1 - `2')) // predicted coverage based on sens and spec - y var for predicated coverage line

	gen x_`i' = (`i'/10) // x var for 45 degree line
	gen y_`i' = (`i'/10) // y var for 45 degree line

	}

	gen id = _n
	keep if id==1 // keep only one observation per by variable

	*reshape new variables to long by id
	reshape long if_ prev_ x_ y_ , i(id)

	*calculate difference between estimated coverage and 45 degree line at true coverage level in population
	gen obsx =  `3' // true coverage in pop
	gen obsy = (`1' * `3') + ((1 - `3') * (1 - `2')) // predicted coverage at true coverage in pop
	replace obsy = `3' if _j<5 // additional y point needed for scatter plot

	*label variables for graph
	label var if_ "Measured Coverage"
	label var x_ "Perfect Measure"
	label var obsy "Difference in Coverage"

	*generate graph
	twoway line x_ y_ , lcolor(gray) lp(dash) || line if_ prev_ , lcolor(red)|| scatter obsy obsx, connect(stairstep) mcolor(red) lc(red) ylabel(0(0.2)1, gmin angle(horizontal)) ytitle("Measured Coverage") ///
		xlabel(0(0.2)1) xtitle("True Coverage")  aspectratio(1) graphregion(color(white)) legend(position(3) cols(1) size(small))

	restore

}
end

********************************************************************************

*PROGRAM: IF_graph_i
*Generates graph of predicted versus true coverage using immediate values
*Requires 3 values to be specified: 1 sensitivity, 2 specificity, 3 true coverage
*Values must be entered in decimal form
*Code example: IF_graph_i 0.8 0.7 0.3

capture program drop IF_graph_i
program define IF_graph_i

quietly {

	preserve

	set obs 1

	*calculate predicted coverage over range of true coverage (0-1) in increments 0.1
	foreach i of num 0/10 {
```

```

    gen prev_`i' = (`i'/10)
    gen if_`i' = ((`i'/10) * `1') + (( 1 - (`i'/10)) * (1 - `2'))

    gen x_`i' = (`i'/10)
    gen y_`i' = (`i'/10)

    }

    gen id = _n
    keep if id==1 // keep only one observation per by variable

    *reshape new variables to long by id
    reshape long if_ prev_ x_ y_ , i(id)

    *calculate difference between estimated coverage and 45 degree line at true coverage level in population
    gen obsx =  `3'
    gen obsy = (`1' * `3') + ((1 - `3') * (1 - `2'))
    replace obsy = `3' if _j<5

    *label variables for graph
    label var if_ "Measured Coverage"
    label var x_ "Perfect Measure"
    label var obsy "Difference in Coverage"

    *generate graph
    twoway line x_ y_ , lcolor(gray) lp(dash) || line if_ prev_ , lcolor(red)|| scatter obsy obsx, connect(stairstep) mcolor(red) lc(red) ylabel(0(0.2)1, gmin angle(horizontal)) ytitle("Measured Coverage") ///
        xlabel(0(0.2)1) xtitle("True Coverage")  aspectratio(1) graphregion(color(white)) legend(position(3) cols(1) size(small))

    restore

}
end

```
